# 3,22-Dioxa-11,14-di­aza­penta­cyclo­[12.8.0.0^2,11^.0^5,10^.0^15,20^]docosa-5(10),6,8,15(20),16,18-hexa­ene-4,21-dione

**DOI:** 10.1107/S1600536813024537

**Published:** 2013-09-12

**Authors:** Xiaolin Ren, Jiao Feng, Jinglin Wang, Bin Liu, Binsheng Yang

**Affiliations:** a Institute of Molecular Science, Chemical Biology and Molecular Engineering Laboratory of the Education Ministry, Shanxi University, Taiyuan, Shanxi 030006, People’s Republic of China; b Department of Chemistry, Changzhi University, Changzhi, Shanxi 046011, People’s Republic of China

## Abstract

In the title compound, C_18_H_14_N_2_O_4_, the piperazine ring adopts a chair conformation and the dihedral angle between the aromatic rings is 13.09 (9)°. In the crystal, mol­ecules are linked along the *c* axis by C—H⋯π and N⋯π [H(N)–centroid distances = 2.8030 (2) and 3.376 (2) Å] inter­actions between neighbouring mol­ecules.

## Related literature

For applications of π–π inter­actions, see: Janiak (2000 ▶). For C—H⋯π inter­actions, see: Ciunik & Desiraju (2001 ▶) and for N⋯π inter­actions, see: Lindeman *et al.* (1998 ▶). For the synthesis of the 2,2′-(ethane-1,2-diylbis(aza­nedi­yl))di­benzoic acid precursor, see: Berger & Telford (2002 ▶).
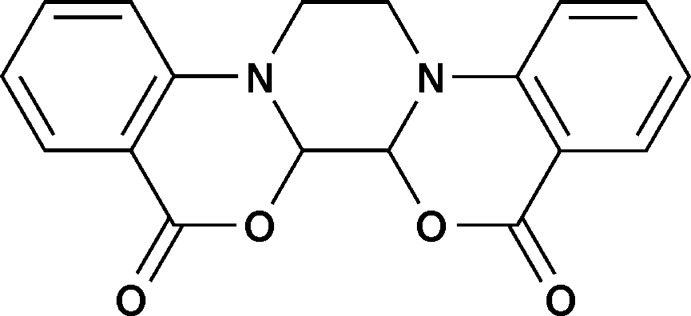


## Experimental

### 

#### Crystal data

C_18_H_14_N_2_O_4_
*M* *_r_* = 322.31
Triclinic, 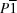

*a* = 8.058 (1) Å
*b* = 8.2629 (11) Å
*c* = 12.0972 (14) Å
α = 74.956 (2)°
β = 73.868 (1)°
γ = 72.311 (1)°
*V* = 723.54 (16) Å^3^
*Z* = 2
Mo *K*α radiation
μ = 0.11 mm^−1^
*T* = 293 K
0.23 × 0.20 × 0.13 mm

#### Data collection

Bruker SMART CCD area-detector diffractometer
Absorption correction: multi-scan (*SADABS*; Bruker, 2005 ▶) *T*_min_ = 0.976, *T*_max_ = 0.986
3858 measured reflections
2533 independent reflections
1301 reflections with *I* > 2σ(*I*)
*R*_int_ = 0.025

#### Refinement

*R*[*F* ^2^ > 2σ(*F* ^2^)] = 0.051
*wR*(*F* ^2^) = 0.098
*S* = 1.01
2533 reflections
217 parameters
H-atom parameters constrained
Δρ_max_ = 0.18 e Å^−3^
Δρ_min_ = −0.19 e Å^−3^

### 

Data collection: *SMART* (Bruker, 1999 ▶); cell refinement: *SAINT* (Bruker, 1999 ▶); data reduction: *SAINT*; program(s) used to solve structure: *SHELXS97* (Sheldrick, 2008 ▶); program(s) used to refine structure: *SHELXL97* (Sheldrick, 2008 ▶); molecular graphics: *SHELXTL* (Sheldrick, 2008 ▶) and *DIAMOND* (Brandenburg, 1999 ▶); software used to prepare material for publication: *SHELXTL* and *PLATON* (Spek, 2009 ▶).

## Supplementary Material

Crystal structure: contains datablock(s) I. DOI: 10.1107/S1600536813024537/ff2118sup1.cif

Structure factors: contains datablock(s) I. DOI: 10.1107/S1600536813024537/ff2118Isup2.hkl

Supplementary material file. DOI: 10.1107/S1600536813024537/ff2118Isup3.cml

Additional supplementary materials:  crystallographic information; 3D view; checkCIF report

## Figures and Tables

**Table 1:** Hydrogen-bond geometry (Å, °) *Cg*1 is the centroid of the C6–C11 ring.

*D*—H⋯*A*	*D*—H	H⋯*A*	*D*⋯*A*	*D*—H⋯*A*
C2—H2⋯*Cg*1^i^	0.98	2.80	3.7337 (3)	159

Symmetry code: (i) 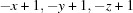.

**Fig. 1.**
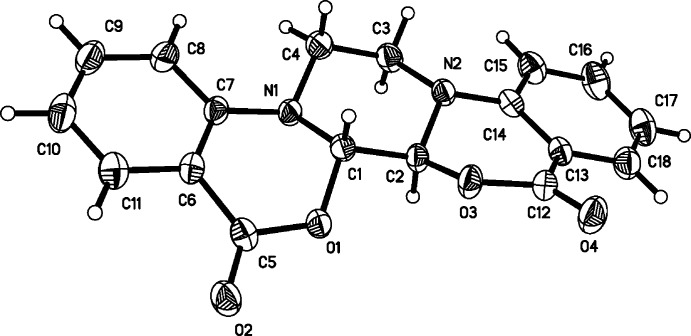
The molecular structure of the title compound with the atom numbering scheme. Displacement ellipsoids are drawn at the 30% probability level. H atoms are presented as small spheres of arbitrary radius.

**Fig. 2.**
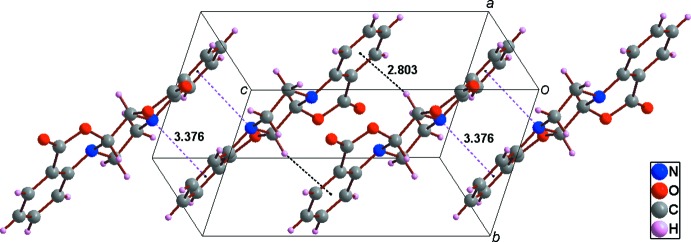
The one-dimensional chain structure of the title compound is formed by N···π interactions (pink dotted lines) and C–H···π interactions (black dotted lines) and extending along the c axis (all distances in Å).

**Fig. 3.**
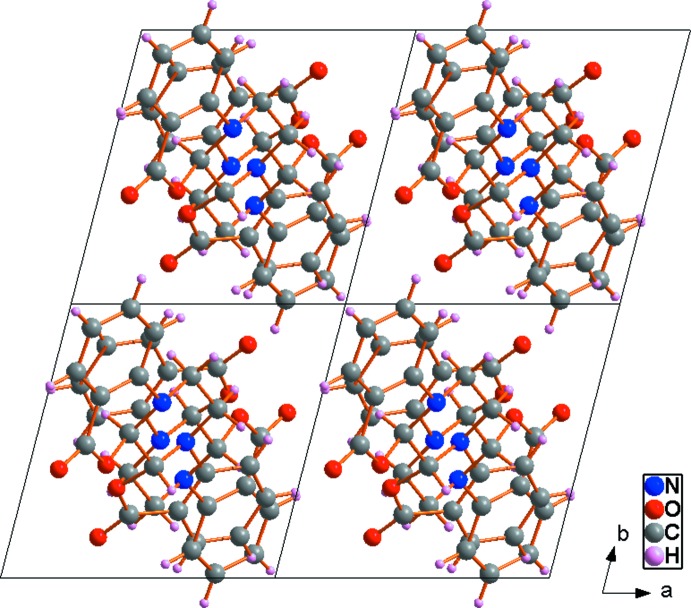
Packing of the title compound viewed along the c axis

## supplementary crystallographic information

### 1. Comment

The C–H···π and π–π interactions are important noncovalent intermolecular forces in determining the crystal packing, molecular assemblies, and structures of large biological systems (Janiak, 2000). In the present work, the crystal of the title compound is generated by noncovalent interactions.

In the title molecule (Fig. 1), the piperazine ring adopts a chair conformation and the dihedral angle between two phenyl rings (C6—C11; C13—C18) is 13.09 (9)°.

As shown in Figure 2, the neighboring molecules of title compound are arranged in a mutual head-to-tail manner by C–H···*Cg*1^i^ (*Cg*1 is the centroid of the C6—C11 benzene ring) interactions (black dotted lines) and N···*Cg*2^ii^ (*Cg*2 is the centroid of the C13—C18 benzene ring) interactions (pink dotted lines)to form infinite one-dimensional chain structure along the *c* axis [symmetry code: (i) 1 - *x*, 1 - *y*,1 - *z*; (ii)1 - *x*, 1 - *y*, 2 - *z*].

The adjacent one-dimensional chains, by van der Waals contacts, stack in a side-by-side fashion along the *c* axis to form three-dimensional structure (Fig. 3).

### 2. Experimental

The precursor 2,2'-(ethane-1,2-diylbis(azanediyl))dibenzoic acid (EDA) was synthesized according to literature procedures (Berger *et al.*, 2002). The title compound was prepared by stirring a methanolic solution of EDA (300 mg, 1.0 mmol) and triethylamine (1 ml) for 10 min at room temperature. Then, 10 ml of a methanol solution containing CuCl_2_·2H_2_O(170 mg, 1 mmol) was added to the mixture and refluxed for 2 h. The mixture was filtered and washed with methanol. The EDA-Cu compound is not achieved as predicted. However, orange single crystals of the title complex suitable for X-ray analysis were obtained after several days from the mother liquor by slow evaporation.

### 3. Refinement

All H atoms were positioned geometrically [C–H = 0.97 Å for CH_2_, 0.93 Å for CH] and refined using a riding model, with *U*iso = 1.2*U*eq of the parent atom.

### Crystal data

**Table:**

C_18_H_14_N_2_O_4_	*Z* = 2
*M**_r_* = 322.31	*F*(000) = 336
Triclinic, *P*1	*D*_x_ = 1.479 Mg m^−^^3^
*a* = 8.058 (1) Å	Mo *K*α radiation, λ = 0.71073 Å
*b* = 8.2629 (11) Å	Cell parameters from 695 reflections
*c* = 12.0972 (14) Å	θ = 2.6–22.8°
α = 74.956 (2)°	µ = 0.11 mm^−^^1^
β = 73.868 (1)°	*T* = 293 K
γ = 72.311 (1)°	Block, orange
*V* = 723.54 (16) Å^3^	0.23 × 0.20 × 0.13 mm

### Data collection

**Table:**

Bruker SMART CCD area-detector diffractometer	2533 independent reflections
Radiation source: fine-focus sealed tube	1301 reflections with *I* > 2σ(*I*)
Graphite monochromator	*R*_int_ = 0.025
phi and ω scans	θ_max_ = 25.0°, θ_min_ = 1.8°
Absorption correction: multi-scan (*SADABS*; Bruker, 2005)	*h* = −7→9
*T*_min_ = 0.976, *T*_max_ = 0.986	*k* = −9→9
3858 measured reflections	*l* = −14→12

### Refinement

**Table:**

Refinement on *F*^2^	Primary atom site location: structure-invariant direct methods
Least-squares matrix: full	Secondary atom site location: difference Fourier map
*R*[*F*^2^ > 2σ(*F*^2^)] = 0.051	Hydrogen site location: inferred from neighbouring sites
*wR*(*F*^2^) = 0.098	H-atom parameters constrained
*S* = 1.01	*w* = 1/[σ^2^(*F*_o_^2^) + (0.0216*P*)^2^] where *P* = (*F*_o_^2^ + 2*F*_c_^2^)/3
2533 reflections	(Δ/σ)_max_ < 0.001
217 parameters	Δρ_max_ = 0.18 e Å^−^^3^
0 restraints	Δρ_min_ = −0.19 e Å^−^^3^

### Special details

**Table:**

Geometry. All e.s.d.'s (except the e.s.d. in the dihedral angle between two l.s. planes) are estimated using the full covariance matrix. The cell e.s.d.'s are taken into account individually in the estimation of e.s.d.'s in distances, angles and torsion angles; correlations between e.s.d.'s in cell parameters are only used when they are defined by crystal symmetry. An approximate (isotropic) treatment of cell e.s.d.'s is used for estimating e.s.d.'s involving l.s. planes.
Refinement. Refinement of *F*^2^ against ALL reflections. The weighted *R*-factor *wR* and goodness of fit *S* are based on *F*^2^, conventional *R*-factors *R* are based on *F*, with *F* set to zero for negative *F*^2^. The threshold expression of *F*^2^ > σ(*F*^2^) is used only for calculating *R*-factors(gt) *etc*. and is not relevant to the choice of reflections for refinement. *R*-factors based on *F*^2^ are statistically about twice as large as those based on *F*, and *R*- factors based on ALL data will be even larger.

### Fractional atomic coordinates and isotropic or equivalent isotropic displacement parameters (Å^2^)

**Table:**

	*x*	*y*	*z*	*U*_iso_*/*U*_eq_	
N1	0.5762 (3)	0.3589 (3)	0.6287 (2)	0.0388 (6)	
N2	0.4532 (3)	0.5027 (3)	0.8359 (2)	0.0397 (6)	
O1	0.7212 (2)	0.5863 (2)	0.54220 (16)	0.0450 (5)	
O2	0.8892 (2)	0.6033 (3)	0.36520 (18)	0.0583 (6)	
O3	0.6612 (2)	0.6710 (2)	0.75579 (16)	0.0481 (5)	
O4	0.6823 (3)	0.8514 (3)	0.85352 (18)	0.0624 (6)	
C1	0.6664 (3)	0.4729 (3)	0.6484 (2)	0.0390 (7)	
H1	0.7706	0.4040	0.6809	0.047*	
C2	0.5451 (3)	0.5917 (3)	0.7294 (2)	0.0388 (7)	
H2	0.4584	0.6804	0.6886	0.047*	
C3	0.3676 (3)	0.3824 (3)	0.8175 (2)	0.0485 (8)	
H3A	0.2659	0.4473	0.7830	0.058*	
H3B	0.3242	0.3144	0.8926	0.058*	
C4	0.4938 (4)	0.2636 (4)	0.7389 (2)	0.0494 (8)	
H4A	0.5862	0.1867	0.7786	0.059*	
H4B	0.4296	0.1932	0.7229	0.059*	
C5	0.8153 (4)	0.5144 (4)	0.4472 (3)	0.0441 (8)	
C6	0.8080 (4)	0.3362 (4)	0.4543 (3)	0.0415 (7)	
C7	0.6848 (4)	0.2634 (3)	0.5421 (3)	0.0398 (7)	
C8	0.6709 (4)	0.1016 (4)	0.5381 (3)	0.0527 (8)	
H8	0.5882	0.0506	0.5955	0.063*	
C9	0.7781 (4)	0.0164 (4)	0.4503 (3)	0.0610 (9)	
H9	0.7666	−0.0916	0.4486	0.073*	
C10	0.9023 (4)	0.0876 (4)	0.3648 (3)	0.0623 (10)	
H10	0.9759	0.0275	0.3065	0.075*	
C11	0.9168 (4)	0.2484 (4)	0.3660 (3)	0.0547 (9)	
H11	0.9993	0.2985	0.3078	0.066*	
C12	0.5914 (4)	0.7686 (4)	0.8402 (3)	0.0468 (8)	
C13	0.4154 (4)	0.7550 (4)	0.9111 (2)	0.0420 (7)	
C14	0.3523 (4)	0.6153 (4)	0.9128 (2)	0.0419 (7)	
C15	0.1977 (4)	0.5903 (4)	0.9944 (3)	0.0554 (9)	
H15	0.1545	0.4958	0.9991	0.066*	
C16	0.1090 (4)	0.7063 (5)	1.0681 (3)	0.0658 (10)	
H16	0.0060	0.6882	1.1226	0.079*	
C17	0.1676 (4)	0.8473 (4)	1.0639 (3)	0.0652 (10)	
H17	0.1038	0.9257	1.1130	0.078*	
C18	0.3217 (4)	0.8707 (4)	0.9860 (3)	0.0561 (9)	
H18	0.3643	0.9648	0.9830	0.067*	

### Atomic displacement parameters (Å^2^)

**Table:**

	*U*^11^	*U*^22^	*U*^33^	*U*^12^	*U*^13^	*U*^23^
N1	0.0462 (14)	0.0398 (14)	0.0361 (15)	−0.0196 (11)	−0.0053 (13)	−0.0104 (12)
N2	0.0460 (14)	0.0456 (15)	0.0318 (15)	−0.0205 (11)	−0.0029 (12)	−0.0100 (12)
O1	0.0564 (13)	0.0462 (12)	0.0346 (13)	−0.0208 (10)	−0.0007 (11)	−0.0122 (11)
O2	0.0621 (14)	0.0704 (16)	0.0456 (14)	−0.0305 (12)	0.0059 (12)	−0.0188 (12)
O3	0.0530 (12)	0.0557 (13)	0.0465 (13)	−0.0281 (10)	0.0007 (11)	−0.0234 (11)
O4	0.0723 (15)	0.0659 (15)	0.0631 (16)	−0.0366 (11)	−0.0022 (12)	−0.0268 (12)
C1	0.0440 (17)	0.0419 (18)	0.0337 (18)	−0.0148 (14)	−0.0061 (15)	−0.0090 (15)
C2	0.0437 (17)	0.0441 (18)	0.0350 (18)	−0.0152 (13)	−0.0070 (15)	−0.0148 (15)
C3	0.0548 (19)	0.058 (2)	0.039 (2)	−0.0284 (16)	−0.0044 (16)	−0.0099 (17)
C4	0.063 (2)	0.0487 (19)	0.044 (2)	−0.0257 (15)	−0.0117 (17)	−0.0083 (17)
C5	0.0393 (18)	0.058 (2)	0.037 (2)	−0.0149 (15)	−0.0047 (16)	−0.0143 (18)
C6	0.0414 (18)	0.0430 (18)	0.044 (2)	−0.0074 (14)	−0.0105 (16)	−0.0172 (16)
C7	0.0449 (18)	0.0387 (17)	0.0404 (19)	−0.0071 (14)	−0.0157 (16)	−0.0125 (16)
C8	0.066 (2)	0.0438 (19)	0.055 (2)	−0.0168 (15)	−0.0167 (18)	−0.0139 (18)
C9	0.077 (3)	0.045 (2)	0.068 (3)	−0.0081 (18)	−0.023 (2)	−0.022 (2)
C10	0.064 (2)	0.059 (2)	0.064 (3)	0.0005 (18)	−0.013 (2)	−0.032 (2)
C11	0.0494 (19)	0.062 (2)	0.053 (2)	−0.0088 (16)	−0.0069 (17)	−0.0213 (19)
C12	0.060 (2)	0.0441 (19)	0.041 (2)	−0.0139 (16)	−0.0116 (17)	−0.0147 (16)
C13	0.0457 (18)	0.0486 (19)	0.0317 (18)	−0.0102 (14)	−0.0056 (15)	−0.0125 (15)
C14	0.0414 (18)	0.052 (2)	0.0321 (18)	−0.0112 (15)	−0.0084 (15)	−0.0083 (16)
C15	0.049 (2)	0.075 (2)	0.047 (2)	−0.0219 (17)	−0.0087 (18)	−0.0140 (19)
C16	0.046 (2)	0.100 (3)	0.051 (2)	−0.0181 (19)	0.0043 (18)	−0.031 (2)
C17	0.060 (2)	0.083 (3)	0.052 (2)	−0.0044 (19)	−0.007 (2)	−0.033 (2)
C18	0.062 (2)	0.056 (2)	0.051 (2)	−0.0088 (17)	−0.0126 (19)	−0.0195 (18)

### Geometric parameters (Å, º)

**Table:**

N1—C7	1.410 (3)	C6—C7	1.385 (4)
N1—C1	1.450 (3)	C6—C11	1.390 (3)
N1—C4	1.456 (3)	C7—C8	1.389 (3)
N2—C14	1.401 (3)	C8—C9	1.371 (4)
N2—C2	1.435 (3)	C8—H8	0.9300
N2—C3	1.459 (3)	C9—C10	1.373 (4)
O1—C5	1.359 (3)	C9—H9	0.9300
O1—C1	1.426 (3)	C10—C11	1.373 (4)
O2—C5	1.199 (3)	C10—H10	0.9300
O3—C12	1.357 (3)	C11—H11	0.9300
O3—C2	1.433 (3)	C12—C13	1.460 (3)
O4—C12	1.208 (3)	C13—C14	1.389 (3)
C1—C2	1.504 (3)	C13—C18	1.391 (3)
C1—H1	0.9800	C14—C15	1.391 (3)
C2—H2	0.9800	C15—C16	1.378 (4)
C3—C4	1.497 (3)	C15—H15	0.9300
C3—H3A	0.9700	C16—C17	1.368 (4)
C3—H3B	0.9700	C16—H16	0.9300
C4—H4A	0.9700	C17—C18	1.367 (4)
C4—H4B	0.9700	C17—H17	0.9300
C5—C6	1.471 (4)	C18—H18	0.9300
			
C7—N1—C1	111.2 (2)	C11—C6—C5	118.5 (3)
C7—N1—C4	117.6 (2)	C6—C7—C8	118.1 (3)
C1—N1—C4	111.3 (2)	C6—C7—N1	118.6 (3)
C14—N2—C2	111.6 (2)	C8—C7—N1	123.2 (3)
C14—N2—C3	117.4 (2)	C9—C8—C7	120.5 (3)
C2—N2—C3	113.7 (2)	C9—C8—H8	119.7
C5—O1—C1	117.6 (2)	C7—C8—H8	119.7
C12—O3—C2	117.6 (2)	C8—C9—C10	121.1 (3)
O1—C1—N1	111.1 (2)	C8—C9—H9	119.4
O1—C1—C2	104.5 (2)	C10—C9—H9	119.4
N1—C1—C2	112.0 (2)	C11—C10—C9	119.4 (3)
O1—C1—H1	109.7	C11—C10—H10	120.3
N1—C1—H1	109.7	C9—C10—H10	120.3
C2—C1—H1	109.7	C10—C11—C6	119.9 (3)
O3—C2—N2	109.6 (2)	C10—C11—H11	120.0
O3—C2—C1	104.6 (2)	C6—C11—H11	120.0
N2—C2—C1	113.3 (2)	O4—C12—O3	117.8 (3)
O3—C2—H2	109.7	O4—C12—C13	126.2 (3)
N2—C2—H2	109.7	O3—C12—C13	116.0 (3)
C1—C2—H2	109.7	C14—C13—C18	120.6 (3)
N2—C3—C4	111.6 (2)	C14—C13—C12	119.4 (3)
N2—C3—H3A	109.3	C18—C13—C12	119.6 (3)
C4—C3—H3A	109.3	C13—C14—C15	118.3 (3)
N2—C3—H3B	109.3	C13—C14—N2	117.9 (3)
C4—C3—H3B	109.3	C15—C14—N2	123.7 (3)
H3A—C3—H3B	108.0	C16—C15—C14	119.6 (3)
N1—C4—C3	111.8 (2)	C16—C15—H15	120.2
N1—C4—H4A	109.3	C14—C15—H15	120.2
C3—C4—H4A	109.3	C17—C16—C15	122.1 (3)
N1—C4—H4B	109.3	C17—C16—H16	119.0
C3—C4—H4B	109.3	C15—C16—H16	119.0
H4A—C4—H4B	107.9	C18—C17—C16	118.7 (3)
O2—C5—O1	117.5 (3)	C18—C17—H17	120.6
O2—C5—C6	126.8 (3)	C16—C17—H17	120.6
O1—C5—C6	115.6 (3)	C17—C18—C13	120.6 (3)
C7—C6—C11	120.9 (3)	C17—C18—H18	119.7
C7—C6—C5	120.4 (3)	C13—C18—H18	119.7
			
C5—O1—C1—N1	−51.0 (3)	C4—N1—C7—C6	−159.6 (2)
C5—O1—C1—C2	−172.0 (2)	C1—N1—C7—C8	152.4 (3)
C7—N1—C1—O1	56.5 (3)	C4—N1—C7—C8	22.5 (4)
C4—N1—C1—O1	−170.4 (2)	C6—C7—C8—C9	0.8 (4)
C7—N1—C1—C2	172.9 (2)	N1—C7—C8—C9	178.7 (3)
C4—N1—C1—C2	−53.9 (3)	C7—C8—C9—C10	0.4 (5)
C12—O3—C2—N2	−49.7 (3)	C8—C9—C10—C11	−1.3 (5)
C12—O3—C2—C1	−171.4 (2)	C9—C10—C11—C6	0.9 (4)
C14—N2—C2—O3	59.1 (3)	C7—C6—C11—C10	0.3 (4)
C3—N2—C2—O3	−165.26 (19)	C5—C6—C11—C10	−174.5 (3)
C14—N2—C2—C1	175.4 (2)	C2—O3—C12—O4	−171.7 (2)
C3—N2—C2—C1	−48.9 (3)	C2—O3—C12—C13	11.6 (4)
O1—C1—C2—O3	−69.7 (2)	O4—C12—C13—C14	−158.6 (3)
N1—C1—C2—O3	169.9 (2)	O3—C12—C13—C14	17.8 (4)
O1—C1—C2—N2	170.9 (2)	O4—C12—C13—C18	14.2 (4)
N1—C1—C2—N2	50.6 (3)	O3—C12—C13—C18	−169.5 (3)
C14—N2—C3—C4	−176.7 (2)	C18—C13—C14—C15	−2.6 (4)
C2—N2—C3—C4	50.3 (3)	C12—C13—C14—C15	170.1 (2)
C7—N1—C4—C3	−174.0 (2)	C18—C13—C14—N2	−179.6 (2)
C1—N1—C4—C3	56.1 (3)	C12—C13—C14—N2	−6.9 (4)
N2—C3—C4—N1	−53.7 (3)	C2—N2—C14—C13	−31.8 (3)
C1—O1—C5—O2	−166.5 (2)	C3—N2—C14—C13	−165.6 (2)
C1—O1—C5—C6	16.3 (3)	C2—N2—C14—C15	151.4 (3)
O2—C5—C6—C7	−164.6 (3)	C3—N2—C14—C15	17.6 (4)
O1—C5—C6—C7	12.2 (4)	C13—C14—C15—C16	1.9 (4)
O2—C5—C6—C11	10.1 (4)	N2—C14—C15—C16	178.7 (3)
O1—C5—C6—C11	−173.0 (2)	C14—C15—C16—C17	0.3 (5)
C11—C6—C7—C8	−1.1 (4)	C15—C16—C17—C18	−1.8 (5)
C5—C6—C7—C8	173.5 (2)	C16—C17—C18—C13	1.1 (5)
C11—C6—C7—N1	−179.2 (2)	C14—C13—C18—C17	1.1 (4)
C5—C6—C7—N1	−4.5 (4)	C12—C13—C18—C17	−171.6 (3)
C1—N1—C7—C6	−29.6 (3)		

### Hydrogen-bond geometry (Å, º)

Cg1 is the centroid of the C6–C11 ring.

**Table:**

*D*—H···*A*	*D*—H	H···*A*	*D*···*A*	*D*—H···*A*
C2—H2···*Cg*1^i^	0.98	2.80	3.7337 (3)	159

Symmetry code: (i) −*x*+1, −*y*+1, −*z*+1.
